# Design of a Dual-Mode Multispectral Filter Array

**DOI:** 10.3390/s23156856

**Published:** 2023-08-01

**Authors:** Zhengnan Ye, Haisong Xu, Yiming Huang, Minhang Yang

**Affiliations:** State Key Laboratory of Extreme Photonics and Instrumentation, College of Optical Science and Engineering, Zhejiang University, Hangzhou 310027, China

**Keywords:** multispectral filter array, multispectral imaging, RGB imaging, spectral sensitivity optimization

## Abstract

Multispectral imaging is valuable in many vision-related fields as it provides an additional modality to observe the world. Cameras equipped with multispectral filter arrays (MSFAs) are typically impractical for everyday use due to their intractable demosaicking and chromatic reproduction processes, which restrict their applicability beyond academic research. In this work, a novel MSFA design is proposed to enable dual-mode imaging for multispectral cameras. In addition to a conventional multispectral image, the camera is also able to produce a Bayer-formed RGB image from a single shot by grouping and merging adjacent pixels in the proposed MSFA, making it suitable for scenarios where display-ready RGB images are required. Furthermore, a two-stage optimization scheme is implemented to jointly optimize objective functions for both imaging modes. The evaluation results on multiple datasets suggest that the proposed MSFA design is able to simultaneously achieve competitive spectral reconstruction accuracy compared to elaborate multispectral cameras and chromatic accuracy compared to commercial RGB cameras.

## 1. Introduction

Spectral imaging systems capture the spectral image of scenes and are widely used in applications such as color reproduction [1,2,3], image enhancement [4,5], object recognition [6,7], and material classification [8,9,10]. Among snapshot spectral imaging techniques, the multispectral filter array (MSFA)-based multispectral camera is rather compact and cost-effective for spectral image acquisition [11]. The camera incorporates a mosaic of pre-determined spectral filters on top of the image sensor to form MSFAs that capture spatial-spectral data in a single shot. However, MSFA filters the spectral data at the pixel level, leading to a trade-off in spatial resolution [12].

For a consumer-friendly camera, it is desirable to have the capability of delivering RGB-colored images. Traditionally, multispectral cameras render the reconstructed spectral image to produce RGB color images. However, this approach is thereby time-consuming, and the color appearance of the rendered RGB image is mostly irrelevant to the original scene. To our knowledge, very few studies have addressed the generation of display-ready RGB images by MSFA.

**Figure 1:**
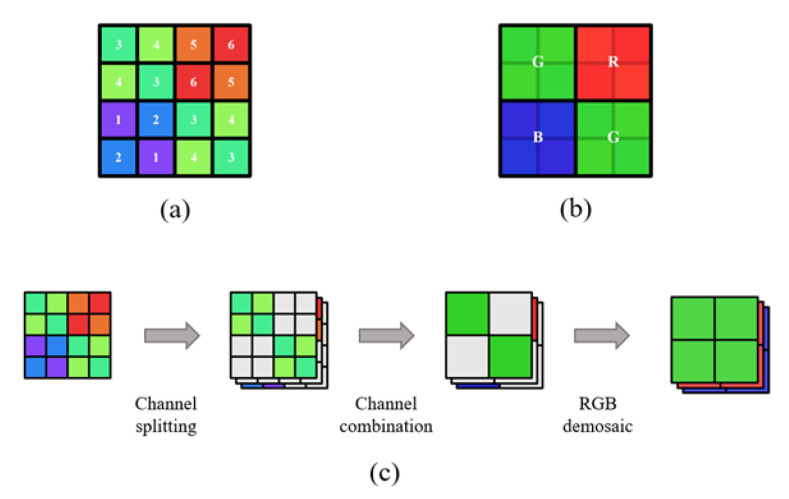
(**a**) The layout of the proposed MSFA; (**b**) The relevant RGB CFA of the MSFA; (**c**) The approach of merging spectral channels in RGB mode.

In this study, we propose an MSFA pattern. The pattern has the potential to simultaneously generate an RGB raw image and a multispectral image. The MSFA comprises six spectral channels for good spectral acquisition accuracy and adopts a channel assignment modeled after the form of Quad-Bayer RGB CFA (color filter array), as illustrated in Figure 1a,b. The spectral channels are divided into three groups, which correspond to the red, green, and blue channels of RGB CFA. The specific MSFA layout enables the generation of a Bayer-form RGB raw image by merging the spectral channels, as shown in Figure 1c. To achieve accurate spectral reconstruction and acceptable RGB image output, we devised a two-stage optimization scheme for the spectral sensitivity functions (SSFs).

**Figure 2:**
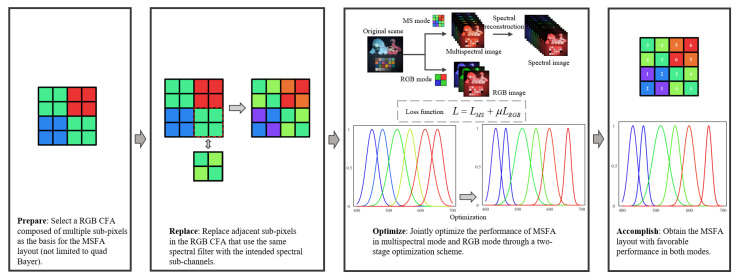
Process of the design of dual-mode MSFA.

The main objectives of this work are summarized as follows:

1. Propose a method to design a dual-mode MSFA pattern, as shown in Figure 2. The method results in an MSFA pattern that enables the camera to output RGB raw images applicable to conventional RGB image signal processing (ISP) pipelines while capturing multispectral images like standard MSFAs;
2. Develop a two-stage optimization scheme consisting of a coarse stage via gradient descent (GD) and a refined stage via particle swarm optimization (PSO) to determine the optimal SSFs;
3. Present an optimal MSFA with (1) competitive accuracy in spectral reconstruction compared to existing MSFAs; and (2) comparable color error in the RGB image to that of commercial RGB cameras.

## 2. Related Works

Early research preferred MSFAs with a series of narrow-band filters for snapshot spectral image capture. As far as we know, Ramanath [13] was one of the earliest to publicly propose the use of MSFA for multispectral image snapshots. In this study, a hexagonal filter arrangement is proposed that includes seven different spectral channels. Some straightforward MSFA layouts are then proposed. Brauers [14] adopted a MSFA by arranging six channels in a 3 × 2 layout to simplify its configuration. Wu [15] placed nine spectral channels, including an infrared channel, into a 3 × 3 MSFA layout to obtain more abundant spectral information. However, the excessive number of channels resulted in a suboptimal spatial sampling rate, which decreased the overall quality of the multispectral images.

Miao [16,17] proposes a generic method for allocating the spectral filters in the MSFA using a binary tree. This method can evenly distribute the pixel positions of each spectral channel. The following studies [18,19] improve its performance by developing corresponding demosaic methods. Several studies [20,21,22,23] then investigate the impact of SSF shape and the number of channels on the binary tree method. The conclusion suggests that 5 to 6 spectral channels are appropriate to balance between spatial and spectral accuracy, and wide-band SSFs have better performance on real-world spectral imaging.

Recently, researchers intend to combine one-shot multispectral imaging technology with RGB imaging. Some studies [24,25,26] review the RGBN filter arrays, in which a near-infrared ray filter replaces one green filter in the conventional Bayer CFA. However, the design of RGBN filter arrays primarily focuses on RGB imaging and performs poorly in multispectral imaging.

Murakami [27] suggests a 4-channel beam splitter with RGB sensors and an extra MSFA sensor, capturing high-resolution RGB images and multispectral images simultaneously. However, the four-sensor framework is extremely complex, and the beam splitter results in low spectral power for the MSFA sensor.

Although there have been extensive studies on optimizing MSFA for spectral reconstruction purposes, there is limited research dedicated to optimizing MSFA for the performance of generating display-ready RGB images. Only a few studies involve RGB applications of MSFA, and they primarily aim to enhance the color capturing capabilities of RGB cameras. The RGB raw images acquired using such designed spectral filter arrays are not compatible with conventional RGB ISP (Image Signal Processing) algorithms.

## 3. Preliminary

### 3.1. Multispectral Response Formation Model

Assuming that a scene is uniformly illuminated and captured by a camera equipped with *C*-channel MSFA, the multispectral image raw response $X\left(u\right)$ at pixel location *u* is given by

(1) $$X\left(u\right)=\sum_{i=1}^{C}\kappa \left(u,i\right)\int_{\Omega }l\left(\lambda \right)\rho \left(\lambda ,U\right)s\left(\lambda ,i\right)\;d\;\lambda$$

where U is the point in the scene corresponding to u. $s\left(\lambda ,i\right)$ is the SSF of the *i*-th channel, $l\left(\lambda \right)$ is the spectral irradiance of the illuminant, $\rho \left(\lambda ,U\right)$ is the spectral reflectance at U, and $\kappa \left(u,i\right)$ is a spatial-dependent binary mask denoting the channel selection of the MSFA pattern, where $\kappa \left(u,i\right)=1$ indicates that the pixel location *u* is assigned to the *i*-th spectral channel and 0 otherwise.

### 3.2. The Dual-Mode Output of Proposed MSFA

To balance spectral accuracy and spatial resolution, we adopt an approach that overlays the sensor with micro-filters of six types of spectral transmittances. As a result, the captured raw image is a single-channel array with a multi-spectral filter pattern. In this pattern, each pixel is only sensitive to a specific band of the spectrum out of the six available. Figure 1a illustrates the proposed MSFA layout, in which the channels are sequentially numbered by their peak wavelengths for clarity.

Similar to the demosaicking procedure in conventional RGB cameras that interpolates 3-channel responses from a single-channel raw array, it is possible to produce a 6-channel multispectral image by interpolating the single-channel raw array from the proposed MSFA with some elaborate demosaic algorithms. We refer to this procedure as “multispectral mode” hereafter.

**Figure 3:**
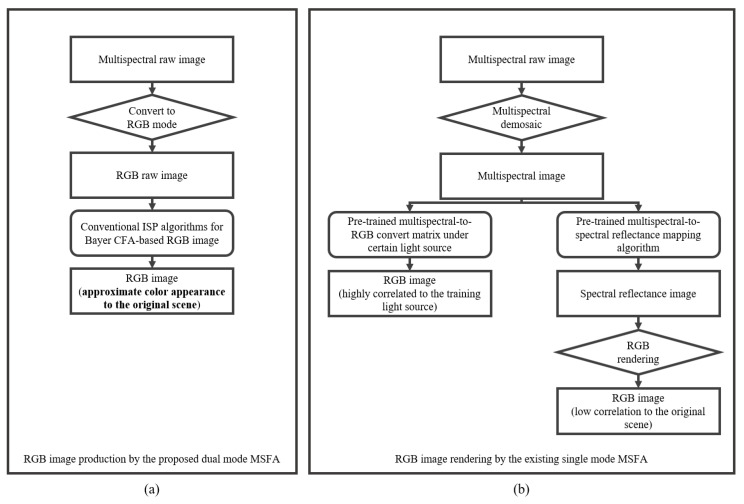
The RGB image processing pipelines of (**a**) the RGB mode of the proposed MSFA and (**b**) the existing single-mode MSFA.

In addition to functioning in multispectral mode, the proposed MSFA can also operate in “RGB mode” because of its innovative micro-filter layout. Taking inspiration from the Quad-Bayer CFA, we group the six types of micro-filters into three categories (R, G, and B) and organize those with comparable transmittance in neighborhoods. We refer to each of these groups as quads, as they consist of 2 × 2 pixels and can be combined into a “big pixel”, as depicted in Figure 1b. In RGB mode, the proposed MSFA generates a Bayer-pattern raw array. Conventional image processing algorithms, such as demosaicking, white balancing, and color correction, can be applied to this raw array to produce an RGB image, as shown in Figure 1c. Figure 3 compares the process of generating RGB images between the RGB mode of the proposed MSFA and the existing single-mode MSFA. It can be observed that the RGB image output of the proposed MSFA has higher fidelity to the original scene.

## 4. Methods

There have been extensive studies optimizing the arrangement of MSFA filters and spectral sensitivity functions, producing high-performance configurations. However, applying the conclusions from these studies directly to a dual-mode MSFA would be inappropriate. This is because the dual-mode MSFA is expected to produce color images in RGB mode. Its equivalent SSFs should be similar to those of a conventional RGB camera, which imposes an additional constraint on the dual-mode MSFA. As a result, it is crucial to develop a new optimization method to improve the performance of the dual-mode MSFA in both RGB and spectral modes. Thus, we developed an objective function that comprises the reconstruction error in both the multispectral and RGB modes. Furthermore, we implemented a two-stage optimization scheme to obtain the optimal configurations for the MSFA. Given that the 6-channel MSFA layout is deterministic, our focus will be on optimizing the SSFs.

### 4.1. Synthetic SSF

As conducted in previous works [14,20], we construct the SSF from some *prior* formation models, in which a set of *K* hyper-parameters ${\left\{P^{k}\right\}}_{k=1}^{K}$ are utilized to control the exact distribution of the SSF

(2) $$s_{i}=f\left(\lambda ;p_{i}^{1},p_{i}^{2},\ldots ,p_{i}^{K}\right)$$

where $i\in \left\{1,2,\ldots ,6\right\}$ is the index of the optimized channels. The choice and the definition of $f\left(\cdot \right)$ will be discussed in Section 5.1.

The SSF matrix of the MSFA is then characterized as

(3) $$S_{P}=f\left(\lambda ,P\right)≜{\left[s_{1},s_{2},\cdots ,s_{6}\right]}^{T}$$

With a slight abuse of notation, we denote the SSF to be optimized as $S_{P}$ hereafter, where ***P*** is the set of optimized hyper-parameters.

### 4.2. Definition of the Training Samples

Two sets of spectral response data, the calibration sample set $R_{calib}$ and the training sample set $R_{train}$, were employed in the optimization scheme according to different computational objectives. $R_{calib}$ is a set of spectral responses, which is used to calculate the pseudo-inverse matrix for spectral reconstruction in the multispectral mode, and the color-correction matrix in the RGB mode. $R_{train}$ consists of small-sized spectral images, referred to as “image blocks”, which are exclusive with $R_{calib}$. The specific composition of the training sample sets will be discussed in Section 5.3.

### 4.3. Objective Function of SSF Optimization

In order to find the optimal SSFs for the proposed MSFA, both the spectral accuracy of the multispectral imaging mode and the color error of the RGB mode should be taken into consideration. For the multispectral mode, the spectral root-mean-square error (RMSE) is chosen to evaluate the spectral reproducibility of the reconstructed spectral images. For the RGB mode, it is more appropriate to evaluate color differences as images captured in this mode are primarily consumed for visual tasks. To this end, we develop an objective function incorporating the two aspects to ensure admirable spectral recovery accuracy and better RGB color reproduction.

#### 4.3.1. Objective Function for Multispectral Mode

For the sake of simplicity, the mosaic and demosaic processes are impermanently disregarded in the following derivation. Under this condition, the captured image is a full-resolution multi-channel image rather than the single-channel raw image mentioned in Section 3.1. The imaging process of the multispectral mode, as presented in Equation (1), can be represented in matrix form as

(4) $$I_{MS}=S_{P}R$$

where $I_{MS}\in R^{6\times N}$ is the output raw array of the multispectral mode, followed by an img2column operator that reshapes a *H* × *W* × 6 image into a 6 × *N* array, where *N* = *H* × *W* is the spatial size of the image. S is the 6 × *M* SSF matrix of the MSFA defined by Equation (3), where *M* is the spectral resolution of SSF. R denotes the *M* × *N* spectral irradiance image of the image scene, which is the element-wise product of the spectral reflectance of the scene and the illuminant spectral power distribution (SPD).

To alleviate the computational burden of optimization iterations, the pseudo-inverse method is employed for spectral reconstruction, in which the spectral irradiance image is reconstructed as

(5) $$R^{\prime }=W\cdot I_{MS}$$

where W is the pseudo-inverse matrix that has been calculated from the calibration set beforehand:

(6) $$\begin{array}{cc} W & =R_{calib}{\left(I_{MS,calib}\right)}^{+}=R_{calib}{\left(S_{P}R_{calib}\right)}^{+}\\ & =R_{calib}{\left(S_{P}R_{calib}\right)}^{T}{\left[S_{P}R_{calib}{\left(S_{P}R_{calib}\right)}^{T}\right]}^{-1}\\ & =R_{calib}R_{calib}^{T}S_{P}{}^{T}{\left(S_{P}R_{calib}R_{calib}^{T}S_{P}{}^{T}\right)}^{-1} \end{array}$$

where $I_{MS,calib}$ is the multispectral responses of $R_{calib}$.

With the pseudo-inverse matrix W, the estimated spectral radiance $R_{train}^{\prime }$ of the training sample set can be obtained by

(7) $$R_{train}^{\prime }=W\cdot I_{MS,train}$$

Finally, the RMSE loss of the multispectral mode can be expressed by the L2-norm of the estimated spectral irradiance $R_{train}^{\prime }$ and the training set $R_{train}$ as

(8) $$\begin{array}{cc} L_{MS} & ={\left\|R_{train}^{\prime }-R_{train}\right\|}_{2}^{2}\\ & ={\left\|W\cdot S_{P}R_{train}-R_{train}\right\|}_{2}^{2} \end{array}$$

#### 4.3.2. Objective Function for RGB Mode

Since the mosaic and demosaic processes are disregarded here, the correlation between RGB images and multispectral images can be simply characterized by a convert matrix:

(9) $$I_{RGB}=M_{MS2RGB}I_{MS}$$

where $I_{RGB}$ is the 3 × *N* RGB response matrix, and $M_{MS2RGB}$ is a 3 *×* 6 convert matrix. Referring to Figure 1a,b, the value of the convert matrix for the proposed MSFA can be derived as

(10) $$M_{MS2RGB}=\left[\begin{array}{cc} \begin{array}{ccc} 0 & 0 & 0\\ 0 & 0 & \frac{1}{2}\\ \frac{1}{2} & \frac{1}{2} & 0 \end{array} & \begin{array}{ccc} 0 & \frac{1}{2} & \frac{1}{2}\\ \frac{1}{2} & 0 & 0\\ 0 & 0 & 0 \end{array} \end{array}\right]$$

For the RGB mode, the standard formula of CIEDE2000 ($\Delta E_{00}$) is adopted to measure the color difference between the estimated RGB values and the ground truth. Accordingly, the device-dependent RGB responses should first be converted into the device-independent CIE1931 XYZ tristimulus for colorimetry calculation, and the correlation of XYZ tristimulus values $I_{XYZ}$ and multispectral image $I_{MS}$ can be given as

(11) $$I_{XYZ}=M_{RGB2XYZ}I_{RGB}=M_{RGB2XYZ}\cdot \left(M_{MS2RGB}I_{MS}\right)=M\cdot I_{MS}$$

where $M_{RGB2XYZ}$ is the 3 × 3 color space conversion matrix, and M is the production of $M_{RGB2XYZ}$ and $M_{MS2RGB}$.

Considering that the ground-truth XYZ tristimulus is obtained by integrating the spectral irradiance with the color mating function of the CIE1931 standard observer, Equation (11) can then be rewritten as

(12) $$\bar{S}R_{calib}=M\cdot I_{MS,calib}=M\cdot \left(S_{P}R_{calib}\right)$$

where $\bar{S}$ is the color matching function of the CIE1931 standard observer.

The convert matrix can then be derived by minimizing the mean-square error between the ground-truth XYZ values and the reconstructed ones via the pseudo-inverse method:

(13) $$\begin{array}{c} M=\bar{S}R_{calib}\cdot {\left(I_{MS,calib}\right)}^{+}=\bar{S}R_{0}\cdot {\left(S_{P}R_{calib}\right)}^{+}\\ =\bar{S}R_{calib}R_{calib}^{T}S_{P}{}^{T}{\left(S_{P}R_{calib}R_{calib}^{T}S_{P}{}^{T}\right)}^{-1} \end{array}$$

The XYZ tristimulus values ${I^{\prime }}_{XYZ,train}$ of the training sample set are then obtained by

(14) $${I^{\prime }}_{XYZ,train}=M\cdot S_{P}R_{train}$$

Subsequently, the loss of the RGB mode is characterized by the CIEDE2000 color difference between the estimated XYZ tristimulus and the ground-truth XYZ tristimulus:

(15) $$\begin{array}{cc} L_{RGB} & =\Delta E_{00}\left({I^{\prime }}_{XYZ,train},\;I_{XYZ,train}\right)\\ & =\Delta E_{00}\left(M\cdot S_{P}R_{train},\;\bar{S}R_{train}\right) \end{array}$$

#### 4.3.3. Overall Objective Function

The overall objective function of the dual-mode MSFA can be constructed by

(16) $$L=L_{MS}+\mu L_{RGB}$$

where μ is an adjustable scale factor to make a tradeoff between two items.

The previous derivations were conducted using full-resolution multispectral images. However, in practical situations, imaging sensors utilize only one microfilter per pixel, resulting in the capture of single-channel raw images. Consequently, the objective function should include functions representing both the mosaic pattern of the filter array and the demosaicking algorithm. With the reintroduction of the mosaic and demosaic processes, Equation (16) can be rewritten as

(17) $$\begin{array}{c} \;L=L_{MS}{}^{\prime }+\mu L_{RGB}{}^{\prime }\\ L_{MS}{}^{\prime }={\left\|W\cdot D_{MS}\left[{ℳ}_{MS}\left(S_{P}R_{train}\right)\right]-R_{train}\right\|}_{2}^{2}\\ L_{RGB}\prime^{}=\Delta E_{00}\left(M\cdot D_{RGB}\left[{ℳ}_{RGB}\left(S_{P}R_{train}\right)\right],\;\bar{S}R_{train}\right) \end{array}$$

where $ℳ\left(\cdot \right)$ and $D\left(\cdot \right)$ denote the mosaic and demosaic algorithms. The guided filter (GF) method [26,28] was adopted for the demosaic of multispectral mode. For lower demosaic error, the third spectral channel, which composes a part of the green channel in the RGB mode with a higher sampling rate, was assigned to be the principal channel in the GF method. On the other hand, for the RGB mode, bilinear interpolation was used for demosaic for the sake of computational convenience.

### 4.4. Two-Stage SSF Optimization

The SSF for the proposed MSFA is optimized in a coarse-to-fine scheme. In the coarse optimization stage, the stochastic gradient descent (SGD) algorithm is implemented to efficiently find a good solution. This solution serves as the initial estimation for the second refined optimization stage. Due to the intrinsic non-differentiability of the CIEDE2000 color difference in Equation (17), which hinders SGD from calculating the gradient of the objective with respect to the hyper-parameters in SSF, we slightly modify Equation (17) and replace the CIEDE2000 metric with a differentiable alternative. The second stage implements a refined optimization with a direct search algorithm to search for the optimal solution in a compact sub-space around the initial estimation from the first stage.

#### 4.4.1. Coarse Optimization

To implement gradient-based optimization algorithms, an end-to-end differentiable model is required. This model builds up a forward graph from the trainable parameters, specifically the hyper-parameters in the SSF formation formula in our case, to the final objective. To this end, we temporarily resort to the L2-norm between the ground truth and the reconstructed spectral irradiance to approximate their CIEDE2000 color difference.

Specifically, in the coarse optimization stage, the hyper-parameters in the SSF are updated in an iterative manner: in each step, the gradient of the objective with respect to the hyper-parameters in the SSF is calculated by chain-rule back propagation, and the hyper-parameters for the next step are updated via the SGD algorithm, as shown in Algorithm 1:
**Algorithm 1:** Stochastic Gradient Descent (SGD)**Input**: Training data $R_{train}$, learning rate *η*, initialization
$P_{0}$ **Output**: Hyper-parameter vector
P $P\leftarrow P_{0}$ **repeat** **forward**: $L\leftarrow L\left(S_{P}\left(\lambda ,P\right),R_{train}\right)$; **backward**: *calculate gradient* ∇L(P) via *backpropagation*; 
**update**: P←P−η∇L(P)

**until** *stopping criterion is not met*;

#### 4.4.2. Refined Optimization

The refined optimization produces the final optimal SSFs $S_{opt}$. It is achieved by updating the hyper-parameters based on the hyper-parameter vector $P_{1}$, which is obtained in the coarse optimization.

Considering the high dimensions of hyper-parameters and the complications in calculating of $\Delta E_{00}$ in Equation (17), a direct search algorithm, the second-order oscillating particle swarm optimization (SOPSO), is adopted for the refined optimization, as shown in Algorithm 2. The algorithm improves the classic PSO by adding a degenerative oscillating term $O\left(\cdot \right)$ to avoid trapping in the local optimum:
**Algorithm 2:** Second-order oscillating particle swarm optimization (SOPSO)**Input**: Training data $R_{train}$, initialization $P_{1}$, max iteration epoch *N*, swarm size *k* **Output**: Hyper-parameter vector
P $x_{i}\leftarrow P_{1}+\varepsilon_{i};P\leftarrow P_{1};L_{best}\leftarrow L\left(S_{P}\left(\lambda ,P_{1}\right),R_{train}\right);\;v_{i}\sim N(0,\varepsilon_{v});E\leftarrow 0;$ **repeat** **for** i∈(1,2,⋯,k); $L_{i}\leftarrow L\left(S_{P}\left(\lambda ,x_{i}\right),R_{train}\right)$;
**end** $L_{iter}\leftarrow \min\left(L_{1},L_{2},\cdots ,L_{k}\right);x_{iter}\sim L_{iter}\left(S_{P}\left(\lambda ,x\right),R_{train}\right)$; **if** $L_{iter}<L_{best}$;
$L_{best}\leftarrow L_{iter};\;P\leftarrow x_{iter}$;
**end** $x_{i}\leftarrow x_{i}+v_{i};v_{i}\leftarrow V\left(v_{i},P\right)\cdot O\left(E\right);E\leftarrow E+1;$ **until** E=N;

## 5. Experiments

### 5.1. Formation of Synthetic SSF

Two types of parameterized SSFs were introduced for optimization. The first is based on the Gaussian function, following previous works [14,15,20]. The SSF of the Gaussian-based spectral channel is defined by two parameters, the central wavelength and the bandwidth, as

(18) $$s_{i}=f\left(\lambda ;P_{i}\right)=f\left(\lambda ;\mu_{i},\sigma_{i}\right)≜e^{-\frac{\lambda -\mu_{i}}{2\sigma_{i}{}^{2}}}$$

where $G\left(\lambda ,\sigma \right)$ denotes the Gaussian function, $\mu_{i}$ and $\sigma_{i}$ are the central wavelength of the *i*-th channel and the bandwidth of the *i*-th channel, respectively. As the default values for the optimization, the central wavelengths of the six channels were uniformly distributed in the visible range, and their bandwidths were assigned to be 40 nm.

The other type is defined by the principal component analysis (PCA) results of a set of real-camera SSFs from Jiang [29], in which the first five components are applied to construct the spectral channel as

(19) $$s_{i}=f\left(\lambda ;P_{i}\right)=f\left(\lambda ;w_{i,1},w_{i,2},\cdots ,w_{i,k}\right)≜\sum_{n=1}^{k}w_{i,n}X_{n}$$

where $X_{n}$ is the *n*-th principal component of the PCA results, and $w_{i,n}$ is the weight of the *n*-th component for the *i*-th spectral channel. To balance the continuity of SSFs and computational complexity, the first five principal components were selected in the optimization process. The default $w_{i,n}$ was assigned to be the average of the weights for the 84 RGB channels in Jiang [29].

### 5.2. Methods for Comparison

To investigate the effect of the dual-mode pattern design on the performance of the MSFA, the single-mode imaging cameras, namely the MSFA-based multispectral cameras and the CFA-based RGB cameras, were adopted for comparisons. The binary-tree-based MSFAs by Monno [20] and Li [21] were taken into consideration as representatives for regularly sampling MSFAs. These two methods differ in the selection of SSFs: the former applies Gaussian-function-based SSFs, and the latter uses SSFs of real commercial filters. The MSFAs by Wu [15] and Brauers [14] were also adopted to show the performance of traditional MSFAs.

For the RGB mode, the SSFs of a Nikon D3X measured in our previous work [30], a Canon 60D, and a SONY NEX-5N measured by Jiang [29] were selected, representing the commercial RGB cameras.

### 5.3. Implementation Details

The gradient calculation and hyper-parameter update in the coarse stage are implemented in Pytorch. The base learning rate for the SGD is set to 2 × 10^−4^, with a weight decay of 10^−9^. We run the training for 500 epochs with the cosine-with-restarts learning rate adjustment scheduler, where the restart cycle and restart gamma are empirically set at 100 and 0.8, respectively.

For the refined stage, the SOPSO runs 30 epochs with a swarm size of 200, and the accelerated coefficients *c*_1_, *c*_2_, and *ω* are set to 1.5, 1.5, and 0.8, respectively.

The optimization was implemented on the ICVL [31] dataset, in which the spectral images were randomly divided into two groups for model training and evaluation, respectively.

The calibration sample set $R_{calib}$ contains 200 pixels randomly selected from the training group. On the other hand, in order to reduce the computational pressure of the cost function, 100 spectral image blocks with 96 × 96 spatial resolution were employed as the training sample set $R_{train}$, which were generated by randomly cropping the spectral images in the training group of the dataset.

Furthermore, the intra-dataset accuracy results of both imaging modes were evaluated by the evaluation group, while the inter-dataset accuracy results were tested on the Harvard [32] dataset.

## 6. Results and Discussion

### 6.1. Optimization Results

**Figure 4:**
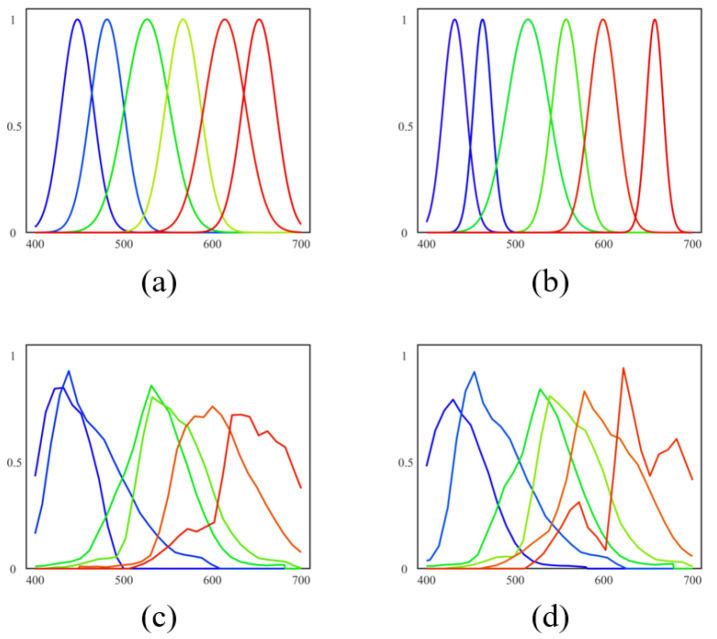
The optimization results for the two types of SSF. The lines denotes the SSFs of six spectral channels. (**a**,**b**) are the initial estimation and final optimum for the Gaussian function-based MSFA, respectively. (**c**,**d**) are the initial estimation and final optimum for the PCA-based MSFA, respectively.

Based on the configuration in Section 5.3, the two-stage optimization scheme was implemented on the ICVL dataset, resulting in the optimal SSFs for the two types of SSFs. The initial estimation from the coarse stage and the final optimal SSFs are shown in Figure 4.

The accuracy results of the optimal dual-mode MSFA camera and the selected multispectral cameras are listed in Table 1. The results indicate that the proposed dual-mode camera achieves comparable accuracy to the single-mode MSFA-based multispectral cameras. Specifically, the Gaussian-based camera performs best in terms of RMSE. This can be attributed to the inclusion of the RMSE-based term in the cost function for the multispectral mode. Similarly, the statistical results of its CIEDE2000 color difference display the highest color accuracy, except for the best 25% section, possibly due to the constraint of RGB mode. The AE values of the proposed MSFA are marginally lower compared to the comparisons, possibly due to the uneven spatial sampling of the proposed MSFA.

It is worth noting that the method proposed by Brauers demonstrates good performance in terms of AE. The MSFA of this method arranges all six spectral channels in a simple side-by-side configuration, resulting in a higher spatial sampling rate compared to other methods, except for the green channel. It is worth mentioning as well that our method using PCA-based SSFs and Li’s method exhibit significantly inferior performance, mainly due to stronger optimization constraints. The PCA-based method derives its SSFs from real camera SSFs, whereas Li’s method selects its SSFs from a set of commercial color filters.

To assess the generalization capability of the dual-mode camera, we evaluated its inter-dataset accuracy using the Harvard dataset, as presented in Table 2. As anticipated, the accuracy of the proposed MSFA experiences a slight degradation, while the Gaussian-based MSFA remains the second-best performer across most metrics. The results suggest that even though the proposed MSFA is designed for dual-mode imaging, its performance in multispectral imaging is still comparable to cameras specialized for dedicated multispectral applications.

Also, we evaluated the RGB image accuracy of the dual-mode MSFA alongside the RGB comparisons. The intra-dataset results are listed in Table 3. It can be observed that the advantages of RGB image accuracy for the proposed method are noteworthy compared to the commercial RGB cameras involved. The only exception is the best 25% section, likely attributed to spectral errors introduced by the direct combination of the subpixels for RGB output. The details of the pixel combination error are discussed in Section 6.2.

Overall, the evaluation results suggest that the dual-mode MSFA exhibits an acceptable level of performance degradation in spectral image reconstruction compared to existing MSFAs specialized for multispectral imaging. Moreover, the RGB mode performs favorably when compared to commercial RGB cameras. The proposed MSFA is verified to have a good balance between multispectral and RGB imaging.

### 6.2. Validity of Channel Combination in RGB Mode

As is revealed above, the pixel combination in the RGB mode introduced an error for the RGB output, where the equivalent SSF of a single RGB channel is constructed by adding two spectral channels together. For example, compared to the ideal green pixel, the subpixels of channels 3 and 4 (referring to Figure 1a) in the proposed MSFA lose the spectral information of channels 4 and 3, respectively, when combined in the RGB mode. This results in differences between the merged RGB pixel response and the ideal RGB response.

**Figure 5:**
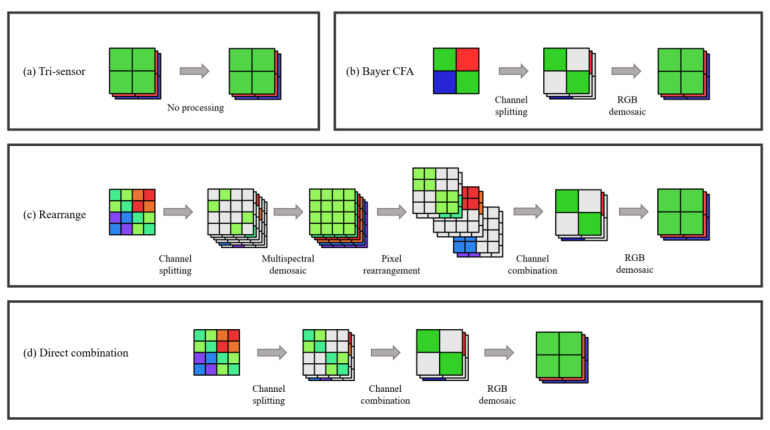
The four compared approaches to delivering full-resolution RGB images. The colored pixels represent specific spectral channels, referring to Figure 1.

Therefore, it is critical to evaluate the actual loss of the pixel combination. The image accuracy of our method was therefore compared with that produced by the conventional RGB CFA. Figure 5 illustrates the different approaches to delivering full-resolution RGB images. Approach (a) is the demosaic-free RGB imaging method, in which every pixel records the responses of the red, green, and blue channels. It is commonly used in tri-sensor RGB cameras that utilize beam splitters. Approach (b) denotes the most commonly used Bayer CFA. The channel SSFs of (a) and (b) are assigned the same as the SSFs of RGB mode by Equation (12). On the other hand, approaches (c) and (d) both denote the RGB output of the dual-mode MSFA. Their difference is that the multispectral channels in (c) demosaiced before pixel combination and then rearranged to produce Bayer CFA-like output, which is illustrated in Figure 5c. Approach (d) is the method we proposed above, which directly combined the channels.

The intra-dataset results, measured by the CIEDE2000 color difference, are presented in Table 4. It is notable that, compared to the common Bayer CFA, the performance of the proposed method has only trivial degradation. This indicates that the direct combination of the spectral channels does not cause unacceptable color errors and is thus appropriate for the RGB image generation of the dual-mode MSFA. Additionally, it is worth mentioning that the rearrange-based method performed slightly better than the Bayer CFA, which might depend on additional spatial information brought in by prepositive multispectral demosaic. Though it demands supplemental computing resources, this method could serve as an optional module for applications that prioritize high accuracy, such as image-based chromaticity measurement and the collection of spectral-RGB image databases.

### 6.3. Performance Degradation in Multispectral Mode

**Figure 6:**
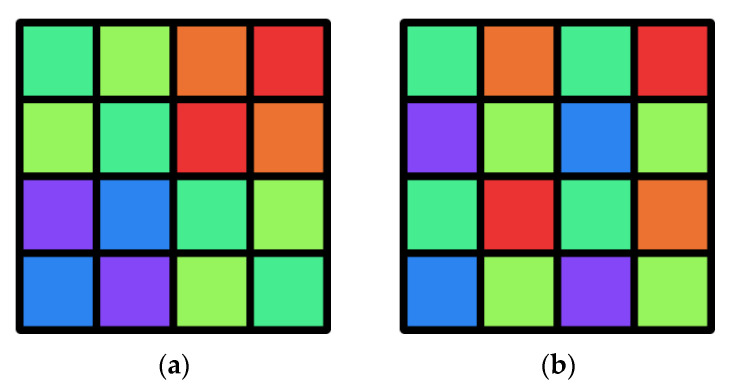
The layouts of (**a**) the proposed MSFA and (**b**) its binary tree-based alternative. The same color in the two layouts represent the same spectral channel.

The layout of the dual-mode MSFA is not optimal for spectral reconstruction compared to the single-purpose MSFAs. This is mainly due to two reasons. Firstly, due to the constraints of the RGB mode, the sampling rates of spectral channels in different spatial directions are not identical in multispectral mode. Secondly, the optimization uses a loss function that includes not only the loss of the multi-spectral mode. In order to examine the impact on spectral reconstruction accuracy due to the ability to generate RGB output, we compared the proposed MSFA with its binary tree-based alternative, which has an equivalent number of channels, as illustrated in Figure 6. An equal number of pixels were assigned to each channel to ensure a reasonable comparison.

Table 5 lists the RMSE results for the comparison. The section “Binary” denotes the results of a binary-tree-based MSFA applying the same SSFs as the proposed MSFA. It is evident that the SSFs are suboptimal for this configuration, as it leads to inferior accuracy results. The section “Binary (opt.)” represents the results of the binary-tree-based MSFA, in which the SSFs are optimized following a similar approach as existing studies on single-mode MSFA optimization. The optimization considered only the spectral error as the loss function, resulting in improved performance. To a certain extent, the performance gap between the optimal binary-tree-based MSFA and the proposed MSFA reflects the trade-off involved in converting a spectral imaging MSFA to a dual-mode MSFA. Nevertheless, given the favorable performance of the dual-mode MSFA, we consider the trade-off to be practical and reasonable. Furthermore, the dual-mode camera can enhance the user friendliness of snapshot multispectral cameras and expand their application range.

## 7. Conclusions

A first-of-its-kind dual-mode MSFA-based multispectral camera is proposed. Besides the ordinary output of a multispectral image, the dual-mode camera is able to generate RGB color images by combining the six spectral channels in pairs. To assess the performance of both modes of the MSFA-based camera, the accuracy of its reconstructed spectral image and RGB image were compared with the existing MSFA-based cameras and commercial RGB cameras, respectively. Evaluation results indicate that both modes of the proposed MSFA achieve comparable performance with the existing methods. Furthermore, two additional experiments were conducted to estimate the accuracy loss associated with dual-mode output. The results suggest that, compared to the optimal MSFA and RGB CFA layouts, the accuracy trade-off of the proposed method is practical and reasonable. The novel design of dual-mode MSFA can enhance the user friendliness of snapshot multispectral cameras and expand their application range.

## Figures and Tables

**Table 1:** The accuracy results of the intra-dataset evaluation for the spectral imaging. The best-performing algorithms are indicated by a green background and bold font, while the second-best are indicated by only bold font.

	RMSE				CIEDE2000				AE			
	Mean	Med.	Best 25%	Worst 25%	Mean	Med.	Best 25%	Worst 25%	Mean	Med.	Best 25%	Worst 25%
Brauers	0.0267	0.0152	0.0078	0.0297	**1.99**	**1.11**	**0.30**	**2.57**	**0.0129**	**0.0071**	**0.0020**	**0.0165**
Wu	**0.0189**	0.0137	0.0078	0.0245	2.55	1.46	0.63	3.21	0.0219	0.0113	0.0045	0.0274
Li	0.0287	0.0166	0.0087	0.0327	3.51	1.92	0.62	4.58	0.0240	0.0139	0.0042	0.0302
Monno	0.0181	**0.0115**	**0.0069**	**0.0219**	2.05	1.15	0.47	2.58	**0.0140**	0.0081	0.0033	**0.0171**
Prop. (PCA)	0.0334	0.0205	0.0102	0.0416	3.46	1.91	0.51	4.63	0.0265	0.0135	0.0036	0.0331
Prop. (Gaussian)	**0.0170**	**0.0112**	**0.0060**	**0.0217**	**1.96**	**1.05**	**0.33**	**2.53**	0.0153	**0.0076**	**0.0021**	0.0188

**Table 2:** The accuracy results of the inter-dataset evaluation for the spectral imaging mode. The best-performing algorithms are indicated by a green background and bold font, while the second-best are indicated by only bold font.

	RMSE				CIEDE2000				AE			
	Mean	Med.	Best 25%	Worst 25%	Mean	Med.	Best 25%	Worst 25%	Mean	Med.	Best 25%	Worst 25%
Brauers	0.0337	0.0252	0.0146	0.0396	2.79	**1.56**	**0.57**	3.83	**0.0174**	**0.0095**	**0.0035**	**0.0228**
Wu	**0.0246**	0.0213	0.0138	**0.0324**	**2.59**	1.60	0.78	**3.45**	0.0214	0.0118	0.0051	0.0267
Li	0.0421	0.0306	0.0178	0.0506	4.53	2.78	1.03	6.34	0.0321	0.0176	0.0065	0.0420
Monno	0.0262	**0.0205**	**0.0125**	0.0333	2.91	1.70	0.69	3.78	**0.0195**	**0.0108**	0.0043	**0.0249**
Prop. (PCA)	0.0502	0.0385	0.0233	0.0625	4.59	2.91	1.09	6.52	0.0349	0.0195	0.0067	0.0460
Prop. (Gaussian)	**0.0256**	**0.0205**	**0.0132**	**0.0330**	**2.72**	1.64	**0.65**	**3.77**	0.0201	0.0111	**0.0040**	0.0259

**Table 3:** The accuracy results of the intra-dataset evaluation for the RGB mode. The best-performing algorithms are indicated by a green background and bold font, while the second-best are indicated by only bold font.

	CIEDE2000				SSIM				PSNR			
	Mean	Med.	Best 25%	Worst 25%	Mean	Med.	Best 25%	Worst 25%	Mean	Med.	Best 25%	Worst 25%
EOS 60D	3.41	1.93	**0.63**	4.49	0.9455	0.9678	0.9862	0.9249	35.64	34.00	39.52	29.70
NEX-5N	3.20	**1.82**	**0.67**	4.15	0.9541	0.9706	0.9882	0.9378	**36.17**	34.51	**39.98**	30.50
D3x	5.36	3.65	1.87	7.17	0.9071	0.9298	0.9704	0.8598	30.54	30.39	34.63	26.31
Prop. (PCA)	**3.11**	1.83	0.86	**3.94**	**0.9575**	**0.9715**	**0.9886**	**0.9420**	35.83	**34.64**	39.79	**30.72**
Prop. (Gaussian)	**2.72**	**1.71**	0.74	**3.42**	**0.9594**	**0.9738**	**0.9893**	**0.9465**	**36.74**	**35.81**	**39.85**	**31.84**

**Table 4:** CIEDE2000 color difference results of the four compared approaches for generating full-resolution RGB images.

	Mean	Med.	Best 25%	Worst 25%
Tri-sensor	1.29	0.91	0.50	1.69
Bayer CFA	2.60	1.65	0.71	3.26
Rearrange	2.57	1.66	0.74	3.24
Direct comb. (proposed)	2.72	1.71	0.74	3.42

**Table 5:** Spectral RMSE results of the proposed MSFA and its binary tree-based alternative.

	Mean	Med.	Best 25%	Worst 25%
Binary	0.0219	0.0153	0.0087	0.0282
Binary (opt.)	0.0135	0.0092	0.0057	0.0177
Proposed	0.0170	0.0112	0.0060	0.0217

## Data Availability

Not applicable.
